# Apolipoprotein B Is Associated With the Microenvironment of Cholangiocarcinoma

**DOI:** 10.3389/fonc.2021.654689

**Published:** 2021-03-31

**Authors:** Xiaofeng Xu, Diyu Chen, Xiaode Feng, Jiating Hu, Jiangzhen Ge, Chaobiao Yan, Deguo Zhang, Zhenan Ling, Jianzhong Chen, Jian Wu

**Affiliations:** ^1^ Division of Hepatobiliary and Pancreatic Surgery, Department of Surgery, The First Affiliated Hospital, Zhejiang University School of Medicine, Hangzhou, China; ^2^ Key Laboratory of Organ Transplantation, Research Center for Diagnosis and Treatment of Hepatobiliary Diseases, Hangzhou, China; ^3^ Institute of Immunology, Zhejiang University School of Medicine, Hangzhou, China

**Keywords:** cholangiocarcinoma, microenvironment, immune, apolipoprotein B, methylation

## Abstract

**Background:**

Cholangiocarcinoma (CCA) is a kind of devastating malignancy, which is correlated with the extremely high mortality. Due to the occult pathogenesis of CCA, most patients are diagnosed in the advanced stage. However, the efficacy of chemotherapy and immunotherapy is limited for these patients. The cause for this phenomenon is unclear, the recent researches indicate that it could be related to predisposing genetic factors and tumor microenvironment (TME) changes. The TME is created by the tumor and dominated by tumor-induced interactions. And the tumor prognosis could be influenced by the extent of infiltrating immune cells and stromal cells in TME.

**Materials and methods:**

The abundance ratio of immune cells for each sample was obtained *via* the CIBERSORT algorithm, and we used ESTIMATE score system to calculate the immune and stromal scores in CCA. The CCA cases in TCGA database were categorized into high and low score groups according to their immune/stromal scores. And then, we identified the differential expressed genes (DEGs) in two groups. Functional enrichment analysis and protein‐protein interaction networks were carried out for DEGs. Interestingly, we found out that apolipoprotein B (APOB) is the most down-regulated among these genes. Then we performed the immunohistochemistry staining of APOB in a CCA tumor microarray which contained 100 CCA cases, APOB was down-regulated in CCA samples. Thus, we evaluated the APOB function in the TME of CCA through TIMER.

**Results and Conclusion:**

The results demonstrate that the infiltration degree of immune cells in CCA could be influenced by the expression of APOB, and the APOB expression could be mediated by DNA methylation. Our study not only indicates APOB is a potential target for CCA immunotherapy but also provides new ideas for researchers to explore the immunotherapy of various tumors.

## Introduction

Cholangiocarcinoma (CCA) is a malignant tumor derived from cells of the biliary tract. CCA presents as several different types of epithelial cell carcinomas, which are characterized by late diagnosis and poor prognosis (1, 2). On the basis of the anatomical locations, CCA could be divided into three different types: intrahepatic cholangiocarcinoma (ICC), perihilar cholangiocarcinoma, and distal cholangiocarcinoma (1, 3, 4). The incidence of CCA is increasing all over the world, and it could be related to an interplay between predisposing genetic factors and environmental triggers (2, 5, 6). Due to the unspecific symptoms, most patients were diagnosed at advanced stages. Chemotherapy and radiotherapy have been poorly effective for patients with inoperable tumors. With the development of further research, immunotherapy has been applied to the treatment of many cancers (4, 7, 8). However, the efficacy of immunotherapy for CCA remains poor, and the mechanism is still unclear.

The tumor microenvironment (TME) refers to the surrounding environment in which tumor cells reside, and consists of fibroblasts, immune cells, glial cells, and extracellular molecules (9, 10). Previous researches have provided the evidence that the TME could influence tumor evolution to a large extent, which subsequently affects tumor metastasis, drug resistance, recurrence, and the prognosis of patients (11–13). Up to now, cancer immunotherapy has attempted to harness the specificity of some important proteins to enhance or restore immune cells in the TME (13, 14). In the TME, immune cells and stromal cells are two major types of non-tumor components and have been proposed to be valuable for diagnostic and prognostic assessment of tumors. To further investigate the role of TME in tumors, a method, referred to as “estimation of stromal and immune cells in malignant tumors using expression data” (ESTIMATE) has been described that can be used to score the stromal and immune fraction in transcriptomic data of cancer tissue (15–17). This algorithm provides elegant analysis on calculating immune and stromal scores to predict the infiltration of non-tumor cells, by analyzing specific gene expression signature of immune and stromal cells, which could help us to discover how the activation of tumor-intrinsic genes shapes tumor microenvironment.

The current study is focused on discovering the potential DNA methylation gene which is associated with the TME changes in CCA. By taking advantages of ESTIMATE algorithm-derived immune scores based on CCA TCGA data sets, a group of TME-associated genes in CCA were extracted. Then we identified that apolipoprotein B (APOB) ranked first in the protein‐protein interaction (PPI) network analysis of the TME-associated genes. Besides, we found out that APOB was down regulated in CCA tissues, and its expression could be modified by DNA methylation. We also validated the immune correlation through the online website “TISIDB,” the data indicated that the infiltration degree of immune cells was related to the DNA methylation of APOB. All these results demonstrated that APOB might act as a potential DNA methylation target for the immunotherapy of CCA. Furthermore, this study also provides a new approach for us to explore immunotherapeutic cell and gene targets for CCA.

## Materials and Methods

### Data Sources

The gene expression data sets analyzed in this study were obtained from the GEO database (https://www.ncbi.nlm.nih.gov/geo/). A total of 1,123 series which were associated with human cholangiocarcinoma were retrieved from the database. After a careful review, specific gene expression profiles, namely, GSE26566 was selected. All of the data utilized in the study are freely available online, and no animal or human experimentation was associated with this study.

### GEPIA

Gene Expression Profiling Interactive Analysis (GEPIA) (http://gepia.cancer-pku.cn/), is a web-based tool with fast and customizable features based on The Cancer Genome Atlas (TCGA) data for analysis of the key interactive gene expression profiles for the TME-associated genes of CCA in this study (18).

### Data Processing of Differential Expressed Genes

We used the GEO2R online analysis tool (https://www.ncbi.nlm.nih.gov/geo/geo2r/) to discover the differential expressed genes (DEGs) associated with the high-immune score/high-stromal score group and the low-immune score/low-stromal score group (high or low grouping is determined based on the median), and the adjusted *P*-values and |logFC| values were calculated. Genes that met the cutoff criteria (adjusted* P* < 0.05 and |logFC|≥2.0), were considered as DEGs. Statistical analyses were carried out for each data set, and the intersecting portions were identified using the Venn diagram webtool (bioinformatics.psb.ugent.be/webtools/Venn/).

### Gene Ontology (GO) and KEGG Pathway Analysis of DEGs

GO analysis is a common but extremely useful method for large scale functional enrichment research; Genes can be classified into different types, namely, genes related to biological processes (BP), genes related to molecular functions (MF), and genes that are cellular components (CC).

KEGG is a database, which collects large amounts of data associated with genomes, biological pathways, diseases, chemical substances, and drugs. The GO annotation analyses and the KEGG pathway enrichment analyses of DEGs involved with this study were performed using the Database for Annotation, Visualization and Integrated Discovery (DAVID) tools (https://david.ncifcrf.gov/). The data sets, which met the cutoff criteria (*P* < 0.01 and gene counts≥10), were considered statistically significant.

### PPI Network Construction and Hub Gene Identification

The DEGs were uploaded to the Search Tool for the Retrieval of Interacting Genes (STRING) database analysis platform to obtain a PPI map. The PPI pairs which possessed a combined score>0.4 were then extracted. Subsequently, the PPI network was visualized with the help of the Cytoscape software (www.cytoscape.org/). The Nodes with higher degrees of connectivity tend to be more essential in maintaining the stability of the entire network. The top 10 genes (ranked according to their centrality indices) were considered to be potential hub gene candidates.

### Immunohistochemistry Assay

The paraffin-embedded 100 CCA (including intrahepatic cholangiocarcinoma (n = 42), extrahepatic cholangiocarcinoma (n = 37), portal cholangiocarcinoma (n = 21)) and 25 adjacent normal liver tissue from our center were used for immunohistochemistry. The tissues were cut at 5 μm, deparaffinized in xylene, and rehydrated in a series of graded alcohol dilutions. Heat epitope retrieval was done for 20 min in a target-retrieval solution in the condition of pH 7.5. The histological sections were incubated with a rabbit polyclonal anti-APOB antibody (Abcam, ID: ab231574) at the dilution of 1:500 overnight at 4°C. Then the slides were incubated with HRP at room temperature for 30 min, and were subsequently visualized using DAB (diaminobenzidine, a chromogenic substance) for 5 to 10 min. The German semi-quantitative scoring system was used to assess staining intensity and area. Each specimen was assigned a score according to the intensity of nuclear staining (no staining/not detected = 0; weak staining/light yellow = 1; moderate staining/yellowish brown = 2; and strong staining/brown = 3) and the fraction of stained cells (0% = 0, 1–24% = 1, 25–49% = 2, 50–74% = 3, and 75–100% = 4). The final score was obtained by multiplying the two scores and ranged from 0 to 12. Low APOB, 0–4; medium APOB, 4–8; high APOB, 8–12. An FSX100 microscope equipped with a digital camera system (Olympus) was used to photograph the specimens.

### University of California Santa Cruz Cancer Genomics Browser Analysis

The University of California Santa Cruz (UCSC) Xena browser (http://xena.ucsc.edu/) was a visualization and analytics tool to analyze and view TCGA database. And UCSC was utilized to access the CCA data in TCGA database. For gene expression, RNA-Seq (polyA+ Illumina HiSeq, n = 36) data were downloaded as log2 (norm_count + 1) values. For the methylation analysis, data from the Illumina Infinium Human Methylation 450 platform were retrieved. This platform expresses DNA methylation as beta values, a continuous variable between 0 and 1 representing the ratio of the intensity of the methylated bead type to the strength of the combined locus (19).

### The Correlation Between Gene Expression and Methylation Around in the Promoter Region

MEXPRESS is a web tool which could offer the visualized analysis of clinical data, the expression (normalized RNASeqV2 value), and methylation TCGA and detect the relationship between them for one single gene in the specific tumor type. In this web tool, it executed the Pearson correlation to evaluate the difference between expression value and methylation level. When MEXPRESS faced with clinical parameter contains only two levels, the system will use a *P* value to compare the difference. The false discovery rate was used to correct for multiple comparisons.

### Statistical Analysis

All the statistical tests and analyses were performed using the SPSS 17.0 software (SPSS) to measure and quantify the relevant parameters associated with the current study. The quantitative data from the control and the experimental groups were compared by using the Student t test or the Wilcoxon signed-rank test. The chi-square test and the Fisher’s exact test were used to evaluate any potential association between APOB expression and the clinic-pathological parameters relevant to the study. The statistical significance level was set at *P < 0.05, **P < 0.01.

## Results

### Immune/Stromal Scores Are Associated With CCA Subtypes and Prognosis

**Figure 1:**
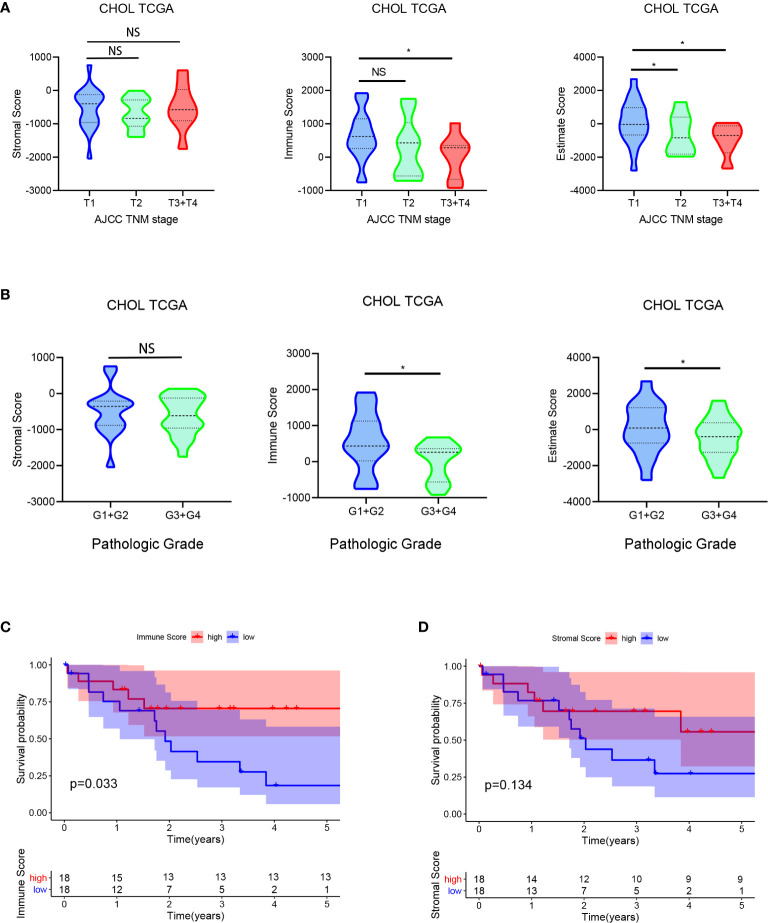
Evaluating immune/stromal scores in cholangiocarcinoma tissues. The obtained CCA RNA-seq profiles were calculated by the ESTIMATE algorithm for immune scores and stromal scores. Then we analyzed the correlation between the immune/stromal scores and clinical characteristics (TNM stage and pathologic grade) **(A, B)**. In addition, based on immune/stromal scores, we divided the CCA patients into high- and low-score groups to investigate the relationship between overall survival and immune/stromal scores **(C, D)**. *P < 0.05.

The 36 cases of CCA RNA-seq profiles and clinical information were obtained from the TCGA database. According to the ESTIMATE algorithm, immune scores ranged from −920.01 and 3550.33, and stromal scores ranged from −2043.23 to 759.51. Then, we investigated the relationship between immune/stromal scores and clinical characteristics to unravel the influence of TME on CCA. Our analysis results in **Figures 1A**, **B** revealed that stromal scores were not correlated with the TNM stage or the pathologic grade. However, the immune scores showed negatively related to TNM stage (*P* = 0.042) and the pathologic grade (*P* = 0.029). Besides, the estimate scores showed correlation to the TNM stage and the pathologic grade (*P* = 0.017, 0.031).

In order to investigate the potential relationship between overall survival and stromal/immune scores, we divided the CCA patients into high-score and low-score groups based on scores and constructed Kaplan-Meier survival curves. We found that the immune score was significantly positively correlated with overall survival (**Figures 1C, D**). Totally, all these results indicated that the immune score was associated with the prognosis of CCA patients.

### Identifying Survival-Related Immune Cells in CCA Microenvironment

**Figure 2:**
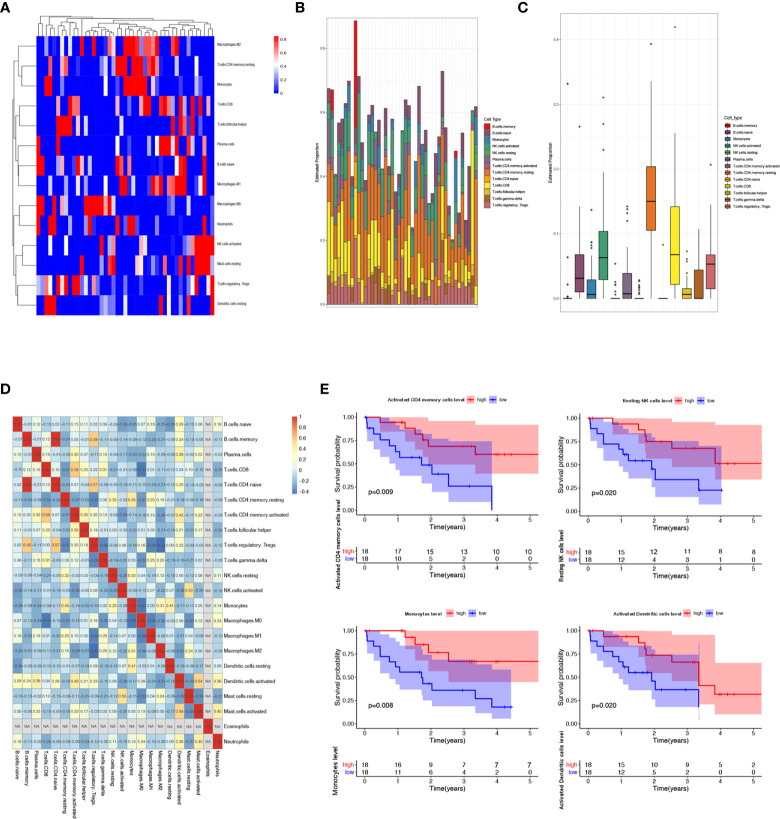
Landscape of immune infiltration in cholangiocarcinoma patients in the TCGA data sets. **(A–C)** Supported evidence indicated that TILs are highly of prognostic and predictive value for various kinds of tumors. According to the CIBERSORT algorithm, the evaluation of the abundance ratio of 22 immune cells in CCA tissues was performed. **(D)** Associated heat map illustrated that B cell memory and T cell CD4 naive were significantly correlated, and macrophage M1 was negatively associated with regulatory T cell in CCA. **(E)** Kaplan-Meier analysis was carried out to further identify the correlation between prognosis value and the 22 types of immune cells abundance ratio. We found out that T cells CD4 memory activated, NK cells resting, monocytes, and dendritic cell abundance ratio were associated to the prognosis of CCA patients.

Increased tumor infiltrating lymphocytes (TILs) are highly of prognostic and predictive value for various kinds of tumors. CIBERSORT, a novel gene expression-based method, can estimate the levels of distinct leukocyte subtypes in tumors (20). Then we performed the CIBERSORT analysis to evaluate the abundance ratio of 22 immune cells in TCGA CCA samples. **Figures 2A–C** showed the infiltration degree of different immune cell subtypes in CCA samples. And compared with normal liver tissues, only 13 immune cell subtypes showed differential infiltration in CCA samples. In addition, as shown in **Figure 2D**, B cell memory and T cell CD4 naive were significantly correlated, and macrophage M1 was negatively associated with regulatory T cell (Tregs). We executed the Kaplan-Meier analysis to further identify the correlation between prognosis value and the 22 types of immune cells abundance ratio. It could be observed that the infiltration level of four immune cells was associated with the overall survival of CCA patients, including T cells CD4 memory activated, NK cells resting, monocytes, and dendritic cells (**Figure 2E**).

### Comparison of Gene Expression Profile With Immune/Stromal Scores in CCA

**Figure 3:**
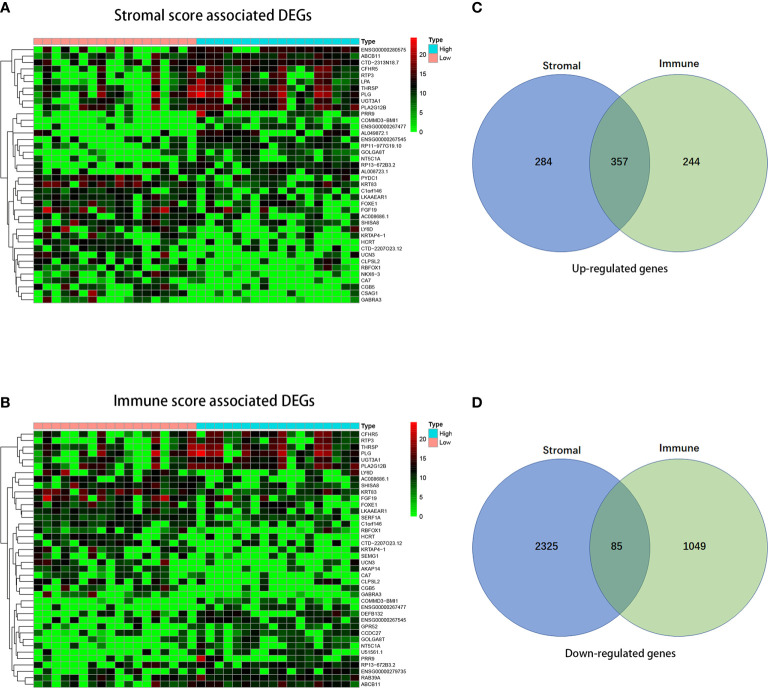
Comparison of gene expression profile with immune scores and stromal scores in cholangiocarcinoma. In accordance with the average linkage method and Pearson distance measurement method, heatmaps were drawn. Red represents higher expressed genes, green represents lower expressed genes. **(A, B)** Heatmap of the DEGs of immune/stromal scores of left panel (low score) vs. right panel (high score). **(C, D)** Venn diagrams showing the number of commonly up-regulated **(C)** or down-regulated **(D)** DEGs in stromal and immune score groups.

To detect the DEGs in high- and low-immune/stromal score groups, we compared 36 CCA gene expression profiles in TCGA database. Heatmaps in **Figures 3A**, **B** showed the differential gene results of the low *vs* high score group (the TOP 40 DEGs were shown in the heatmap). Based on stromal scores, we compared the high score group to low score group and found out that 641 genes were up-regulated and 2,410 genes were down-regulated. In comparison to the low immune score group, 601 genes were up-regulated and 1134 genes were down-regulated in the high immune score group (**Figures 3C, D**). Besides, the Venny diagram demonstrated that 357 DEGs were up-regulated, and 85 DEGs were down-regulated both in stromal/immune high score groups. Therefore, we decided to concentrate on the DEGs mentioned above for the subsequent analysis.

### Functional Enrichment Analysis of DEGs in CCA Microenvironment

**Figure 4:**
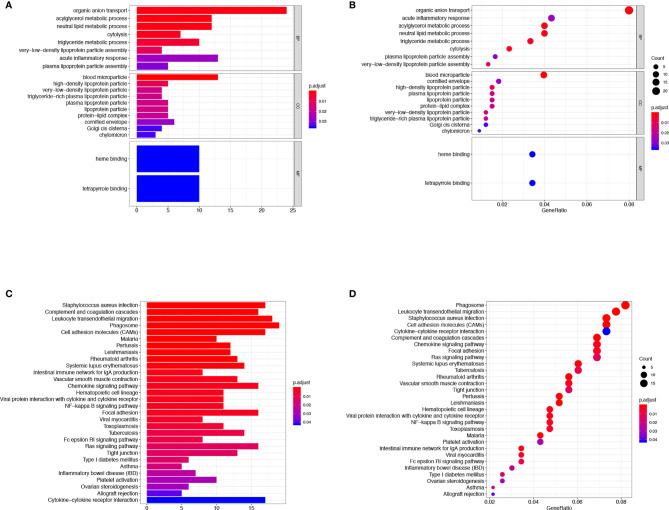
GO term and KEGG pathway analysis for DEGs significantly associated with TME infiltration in ICC. The GO functional clustering and KEGG pathway enrichment analysis were carried out utilizing the DAVID. The results of the GO **(A, B)** and the KEGG pathway enrichment analyses **(C, D)** for the identification of DEGs in CCA.

Then we evaluated the biological functions of these DEGs in CCA, we performed the GO functional clustering and KEGG pathway enrichment analysis by DAVID. A total of 20 GO terms of biological process were identified (false discovery rate, or FDR < 0.05, -log FDR> 1.301). The results showed that the DEGs were mainly enriched in the biological process, including organic anion transport, acylglycerol metabolic process, neutral lipid metabolic process, cytolysis, triglyceride metabolic process, and acute inflammatory response. Furthermore, 10 terms of cellular component were discovered, such as blood microparticle, high-density lipoprotein particle, and very-low-density lipoprotein particle (**Figures 4A, B**). KEGG pathway analysis (**Figures 4C, D**) demonstrated that the leukocyte transendothelial migration, chemokine signaling, NF-κB signaling pathway, inflammatory bowel disease, and cytokine-cytokine receptor interaction, etc, were influenced by these DEGs. In brief, all these DEGs were related to the transmission of various signaling pathways which were associated with immune cell infiltration.

### Protein-Protein Interaction Network Construction, Hub Genes and Module Analysis

**Figure 5:**
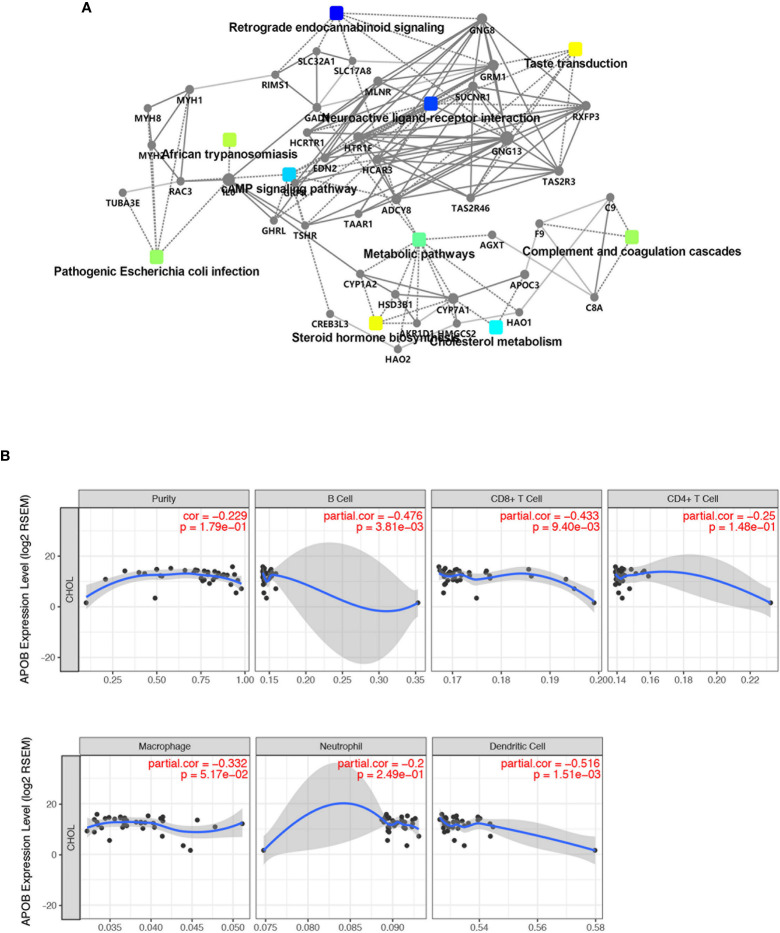
The expression of APOB was related to the immune cell infiltration in CCA. **(A)** The probability of relationship between pathways of DEGs was evaluated by STRING tools. The PPI network was established. **(B)** TIMER database was used to validate the correlation between APOB expression and the level of immune cell infiltration in CCA. Followed by the up-regulation of APOB, the purity of B cells, CD8+ T cells, CD4+ T cell, macrophage, neutrophil, dendritic cells were reduced.

As shown in **Figure 4**, 30 terms for KEGG pathway enrichment analysis were significantly over-represented among these TME DEGs in CCA samples. Then, the probability of relationship between pathways was evaluated by STRING tools. The PPI network was presented in **Figure 5A**. The co-expression network genes were mainly related to various signaling pathways associated with cancer, including retrograde endocannabinoid signaling, cholesterol metabolism, and cAMP signaling pathway. The top 10 hub genes in PPI network was represented in **Table 1**, including the gene symbol and full gene names. Then, the 10 hub genes were apolipoprotein B (APOB, degree = 30), interleukin 6 (IL6, degree = 30), apolipoprotein C3 (APOC3, degree = 24), haptoglobin (HP, degree = 21), histidine-rich glycoprotein (HRG, degree = 20), complement C8 alpha chain (C8A, degree = 18), fibrinogen gamma chain (FGG, degree = 17), apolipoprotein A5 (APOA5, degree = 16), coagulation factor IX (F9, degree = 14), and plasminogen (PLG, degree = 13).

**Table 1:** Top 10 Hub Genes With Higher Degree Of Connectivity.

Gene Symbol	Gene Description	Degree
APOB	Apolipoprotein B	30
IL6	Interleukin 6	30
APOC3	Apolipoprotein C3	24
HP	Haptoglobin	21
HRG	Histidine-rich glycoprotein	20
C8A	Complement C8 alpha chain	18
FGG	Fibrinogen gamma chain	17
APOA5	Apolipoprotein A5	16
F9	Coagulation factor IX	14
PLG	Plasminogen	13

To further clarify the expression of these hub genes in CCA and normal tissues, we performed the differential expression analysis by using GEPIA tools. The results From the box-profiles in **Figure S1** indicated that APOB, APOC3, HP, HRG, FGG, APOA5, F9 and PLG were significantly down-regulated in CCA tissues compared to normal liver tissues. Combining all these results, we thought APOB might play an important role in CCA microenvironment.

### APOB Is Down-Regulated and Associated With CCA Immune Cells Infiltration

In order to prove the expression pattern of APOB in CCA, we observed that APOB was down-regulated in CCA samples compared to normal liver samples and bile tract samples. All these results indicated that APOB might act as a suppressor gene in CCA (**Figures S2A, B**). In order to further evaluated the correlation between the APOB and clinical characteristics, then we performed the immunohistochemistry staining of APOB in a CCA tumor microarray (TMA), which contained 100 CCA cases from our center. As **Figures S2C**, **D** showed, about 53% of the cases presented the low expression of APOB, and 29% and 18% of the CCA tissues showed the medium and high expression of APOB respectively. Then, based on the TNM stage (American Joint Committee on Cancer (AJCC), 2017 8th) level, the CCA tissues were divided into T1+T2 group and T3+T4 group, our results revealed that the APOB expression level in T3+T4 group was lower than that in T1+T2 group. Besides, we also identified that, followed by the increase of pathological grade, the expression of APOB reduced (**Figure S2E**, *P* < 0.05). Through analyzing these results mentioned above, APOB would be a core target in CCA microenvironment, thus evaluating the relationship and interaction with immune cells are of great value for further immune-related research. In this study, we used TIMER database to validate the correlation between APOB expression and the level of immune cell infiltration (**Figure 5B**). We identified that, followed by the up-regulation of APOB, the purity of B cells, CD8+ T cells, CD4+ T cell, macrophage, neutrophil, dendritic cells were reduced.

### APOB Expression Is Associated With Immune-Associated Pathways in CCA

**Figure 6:**
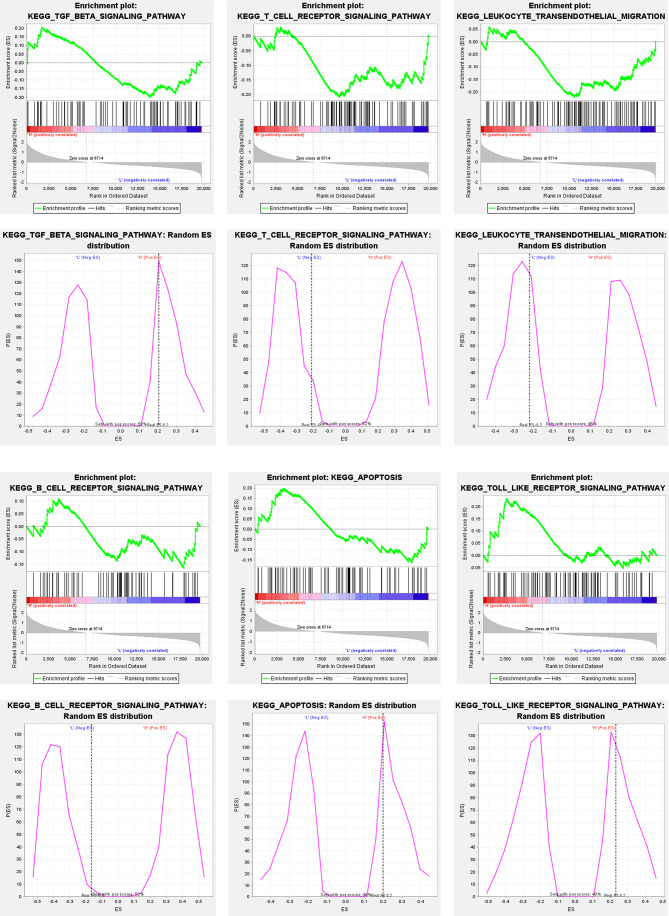
GSEA for CCA cases in GSE26566 based on the level of APOB expression. It was observed that the TGF- β signaling pathway, T cell receptor signaling pathway, leukocyte transendothelial migration, B cell receptor signaling pathway, apoptosis pathway, and Toll like receptor signaling pathway were significantly enriched.

To explore the relevance of APOB expression to CCA microenvironment, we carried out Gene Set Enrichment Analysis (GSEA) for CCA cases in GSE26566 based on the level of APOB expression. As **Figure 6** showed, the TGF- β signaling pathway, T cell receptor signaling pathway, leukocyte transendothelial migration, B cell receptor signaling pathway, apoptosis pathway, and Toll like receptor signaling pathway were significantly enriched. These involved signaling pathways all acted as the important roles in tumor immune response for CCA TME.

### DNA Methylation Level of APOB Would Impact CCA Microenvironment

**Figure 7:**
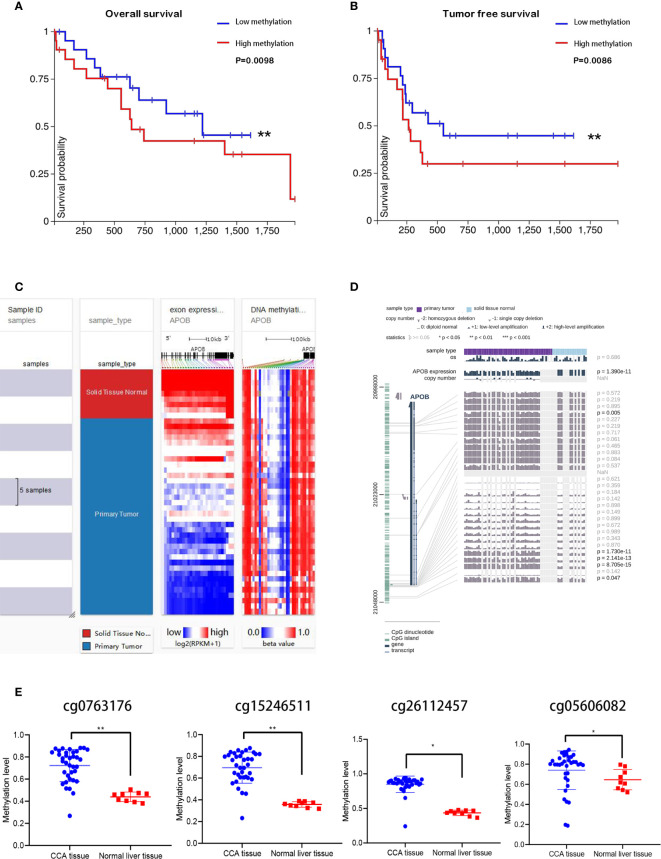
The APOB expression was correlated to DNA methylation in CCA. Through Kaplan-Meier analysis, the high APOB DNA methylation group showed worse prognosis than low APOB DNA methylation group in CCA **(A, B)**. Taking the application of the UCSC Xena database, we observed that normal liver tissues showed higher APOB expressions than CCA tissues, and the DNA methylation level was negatively correlated with the APOB expression in CCA **(C)**. Additionally, with the help of MEXPRESS, it can be noted that the tumor tissues showed higher methylation levels when compared with the normal liver tissues in the APOB CpG islands—cg0763176, cg15246511, cg26112457, and cg05606082, these four sites are the target sites of the APOB CpG island **(D, E)**. *P < 0.05, **P < 0.01.

As a major epigenetic modification method, DNA methylation is implicated in various biological process, which acts as a gene silencing mechanism, and the CpG island in gene promoter regions is prone to methylation for a variety of reasons. In this study, relationship between the APOB and DNA methylation in CCA was investigated. The **Figures 7A**, **B** depict our findings that compared to the patients with low levels of APOB DNA methylation, the patients with high DNA methylation levels of the APOB gene were associated with a worse overall survival and tumor free survival rate among the patients with CCA. We found that the methylation of APOB impacted the prognosis of CCA patients. Taking the application of the UCSC Xena database, we observed that normal liver tissues showed higher APOB expressions than CCA tissues, and the DNA methylation level was negatively correlated with the APOB expression in CCA (**Figure 7C**).

Next, we used Meth Primer to study and identify methylation sites in the APOB CpG island. It was found out that there were three methylation sites in APOB CpG island. In addition, we analyzed CCA MEDIP sequence data collected from TCGA through MEXPRESS database. The results and observations of these analyses are shown in **Figures 7D**, **E**, and from the results it can be noted that the tumor tissues showed higher methylation levels when compared with the normal liver tissues in the APOB CpG islands—cg0763176, cg15246511, cg26112457, and cg05606082, these four sites are the methylation target sites of the APOB CpG island. All these results indicate that the down-regulation of APOB in CCA is associated with promoter hyper-methylation. Subsequently, the 36 CCA cases were divided into APOB low/high DNA methylation group based on the MEDIP sequence data. The results in **Figure S3** showed that followed by the increase of APOB DNA methylation level, the infiltration of neutrophils and B cell naive were accelerated, but the infiltration of regulatory T cells and CD8+ T cells were inhibited. On the whole, all these results indicated that DNA methylation level of APOB might have the influence on the CCA microenvironment.

## Discussion

Cholangiocarcinoma consists of a variety of heterogeneous carcinomas with characteristics of biliary tract differentiation, and is thought to arise from the intrahepatic or extrahepatic biliary tract (3, 21). The etiology might be related to bile duct stones, primary sclerosing cholangitis (PSC) and other diseases (7, 22). Up to now, surgery, radiotherapy, and chemotherapy are used to treat the CCA patients, but the curative effect is limited (23). With the development of research, immunotherapy has been applied to the treatment for many kinds of tumor. In addition, many studies have found that tumor microenvironment (TME) plays an important role in tumor immunotherapy response.

The tumor microenvironment is a vascular network, which consists of different kinds of cells, including fibroblasts, immune cells, and stromal cells (9, 24). Several pieces of evidence have indicated TME was not only associated with tumor initiation, progression, and metastasis, but also could influence the therapeutic response and clinical outcome of various tumors (24, 25). The interactions between TME and tumor cells promoted a portion of tumor cells escaping from the immunological surveillance and accelerated the progression of tumors. According to the heterogeneity of TME at various stages of tumorigenesis, microenvironment-targeted therapy strategies were proposed, such as blocking the extracellular ligand-receptor interactions and correlated signaling pathways (26). Up to now, the correlation between the CCA and TME was still unknown. Therefore, it is necessary to discover the regulatory mechanism of microenvironment in CCA.

In this study, utilizing the CIBERSORT algorithm, we estimated the infiltration level of each immune cell subtype of each CCA specimen obtained from TCGA database. Through the integrative analysis, our findings revealed that the abundance ratio of the four immune cells was related to the survival of CCA patients *via* Kaplan-Meier analysis, including T cell CD4 memory activated, monocyte, NK cells resting, and dendritic cells activated. Then we also calculated the immune and stromal scores of these CCA samples through ESTIMATE score system. And 442 differentially expressed genes (DEGs) were yielded from comparison of high vs. low immune scores (or stromal scores) groups. Then we further identified the hub genes among these DEGs using the PPI network analysis. Interestingly, it was found out that APOB ranked top 1 in the PPI network, and the GEO database also confirmed that APOB was significantly downregulated in the CCA TME. Thus, we suspected that APOB might be associated with the immune cell infiltration in CCA.

**Figure 8:**
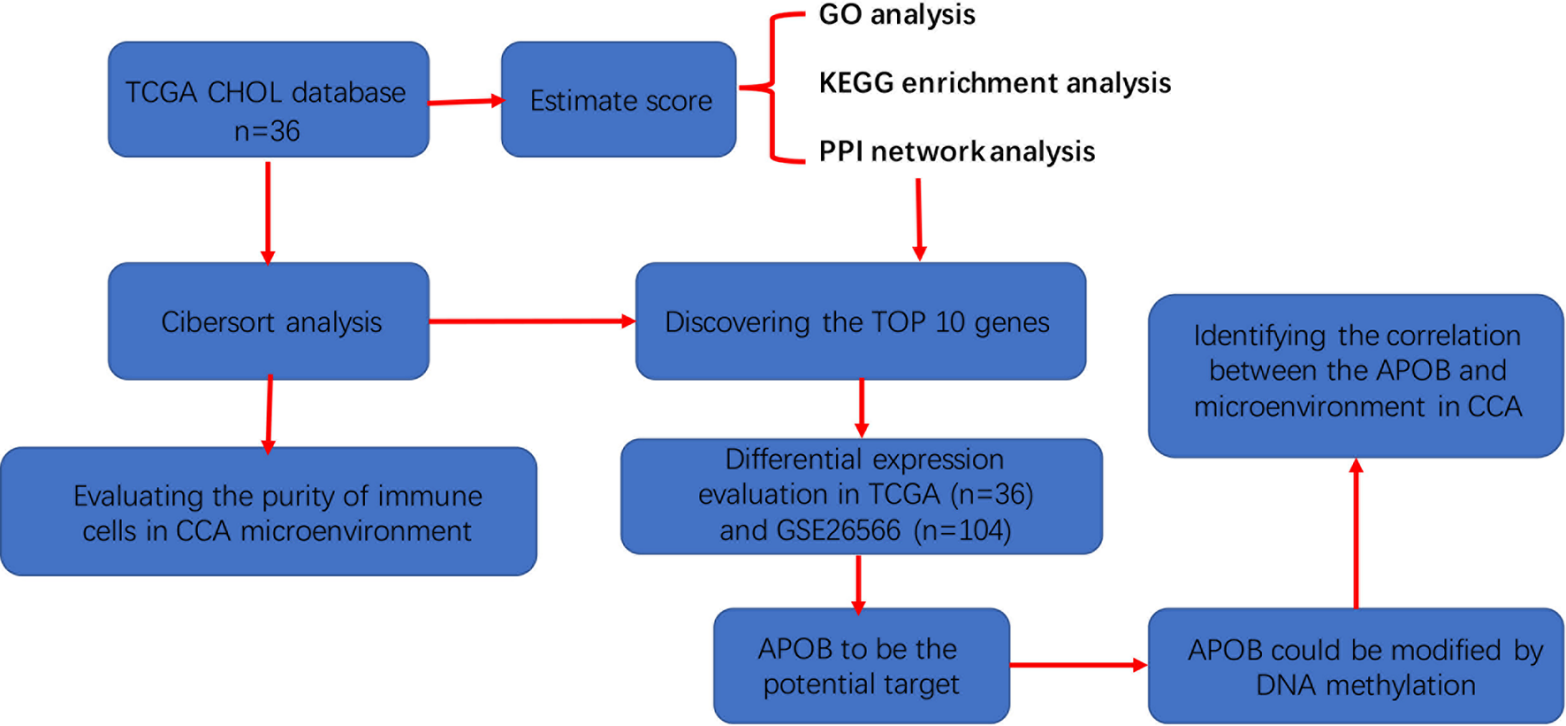
The detailed flowchart of this study to discover the role of APOB in the microenvironment of cholangiocarcinoma.

APOB is known to be a member of the apolipoprotein family, but in this study, we firstly revealed that it contributed to the TME establishment of CCA. The research strategy was presented in **Figure 8**. Based on the evidence mentioned above, we used TIMER database to validate the correlation between APOB expression and the level of immune cell infiltration. We identified that the purity of B cells, CD8+ T cells, CD4+ T cells, macrophage, neutrophil, dendritic cells were reduced followed by the up-regulation of APOB in CCA. Besides, GSEA were also carried out for CCA cases in GSE26566 based on the expression of APOB. As the results shown, tumor immune response involved pathways, such as TGF-β signaling pathway, T cell receptor signaling pathway, and leukocyte trans-endothelial migration pathway were changed followed by the differential expression of APOB in CCA. From the TCGA database of CCA, we found that the tumor tissues showed higher DNA methylation levels when compared to their normal liver tissue at the sites cg0763176, cg15246511, and cg26112457, and cg05606082 - which were the target sites of the APOB CpG island. It can therefore be suggested that APOB could be modified by DNA methylation and associated with the microenvironment reprogramming in CCA tissues.

APOB has been confirmed by many studies to be a gene closely related to lipid metabolism and can play an important role in atherosclerosis, non-alcoholic fatty liver, and cerebrovascular stroke. Reviewing the literatures, we found that APOB played a role as an oncogene in many tumors, but not as a potential tumor suppressor gene in cholangiocarcinoma as we found. Whether APOB makes a special contribution in the pathogenesis of CCA, it should be further explored in the future. Besides, we identified that DNA methylation mediated the downregulation of APOB in CCA. And the DNMTs inhibitor-5-Aza has been used in the treatment of glioma and lung adenocarcinoma. Therefore, we also should investigate the function of 5-Aza in CCA, it might reveal a novel therapeutic method for the CCA treatment.

In summary, we identified that the infiltration degree of immune cells in CCA could be influenced by the expression of APOB. Our study not only indicates that APOB is a potential target for CCA immunotherapy, but also provides new ideas for researchers to explore the immunotherapy of various tumors.

## Data Availability Statement

The data sets presented in this study can be found in online repositories. The names of the repository/repositories and accession number(s) can be found in the article/**Supplementary Material**.

## Ethics Statement

The studies involving human participants were reviewed and approved by the Ethics Committee of the First Affiliated Hospital of Zhejiang University School of Medicine. The patients/participants provided their written informed consent to participate in this study.

## Author Contributions

XX: study design and data analysis. DC: study design and experimental operation. XF: experimental operation and data analysis. JH: specimen collection and statistics. JG: specimen collection. CY: specimen collection and experimental operation. DZ: experimental operation. ZL: experimental operation. JC: program guidance and supervision. JW: program guidance and supervision. All authors contributed to the article and approved the submitted version.

## Funding

This study was supported by the National Natural Science Foundation of China (81874228, 82073144, and 81800558) and Grant from Health Commission of Zhejiang Province (JBZX-202004).

## Conflict of Interest

The authors declare that the research was conducted in the absence of any commercial or financial relationships that could be construed as a potential conflict of interest.

## Supplementary Material

Supplementary Figure 1 Using GEPIA tools, these results indicated that APOB, APOC3, HP, HRG, FGG, APOA5, F9, and PLG were significantly down-regulated in CCA tissues compared to normal liver tissues.

Supplementary Figure 2
**(A, B)** According to the GSE26566, we identified APOB was down-regulated in CCA tissues compared to the normal liver tissues counterparts. **(C, D)** The immunohistochemistry staining of APOB was performed in the CCA tumor microarray, about 53% of the cases presented the low expression of APOB, and 29% and 18% of the CCA tissues showed the medium and high expression of APOB respectively. **(E)** based on the TNM stage level, our results revealed that the APOB expression level of the CCA tissues in T3+T4 group was lower than that in T1+T2 group. Besides, we also identified that, followed by the increase of pathological grade, the expression of APOB reduced.

Supplementary Figure 3 Based on the MEDIP sequence data, the results showed that followed by the increase of APOB DNA methylation level, the infiltration of neutrophils and B cell naive were accelerated, but the infiltration of regulatory T cells and CD8+ T cells were inhibited in CCA.
