# Supplementary figures and images for: Apolipoprotein B Is Associated With the Microenvironment of Cholangiocarcinoma
Source: Front Oncol. 2021 Mar 31;11:654689. doi: 10.3389/fonc.2021.654689 (PMC8092120; doi:10.3389/fonc.2021.654689)

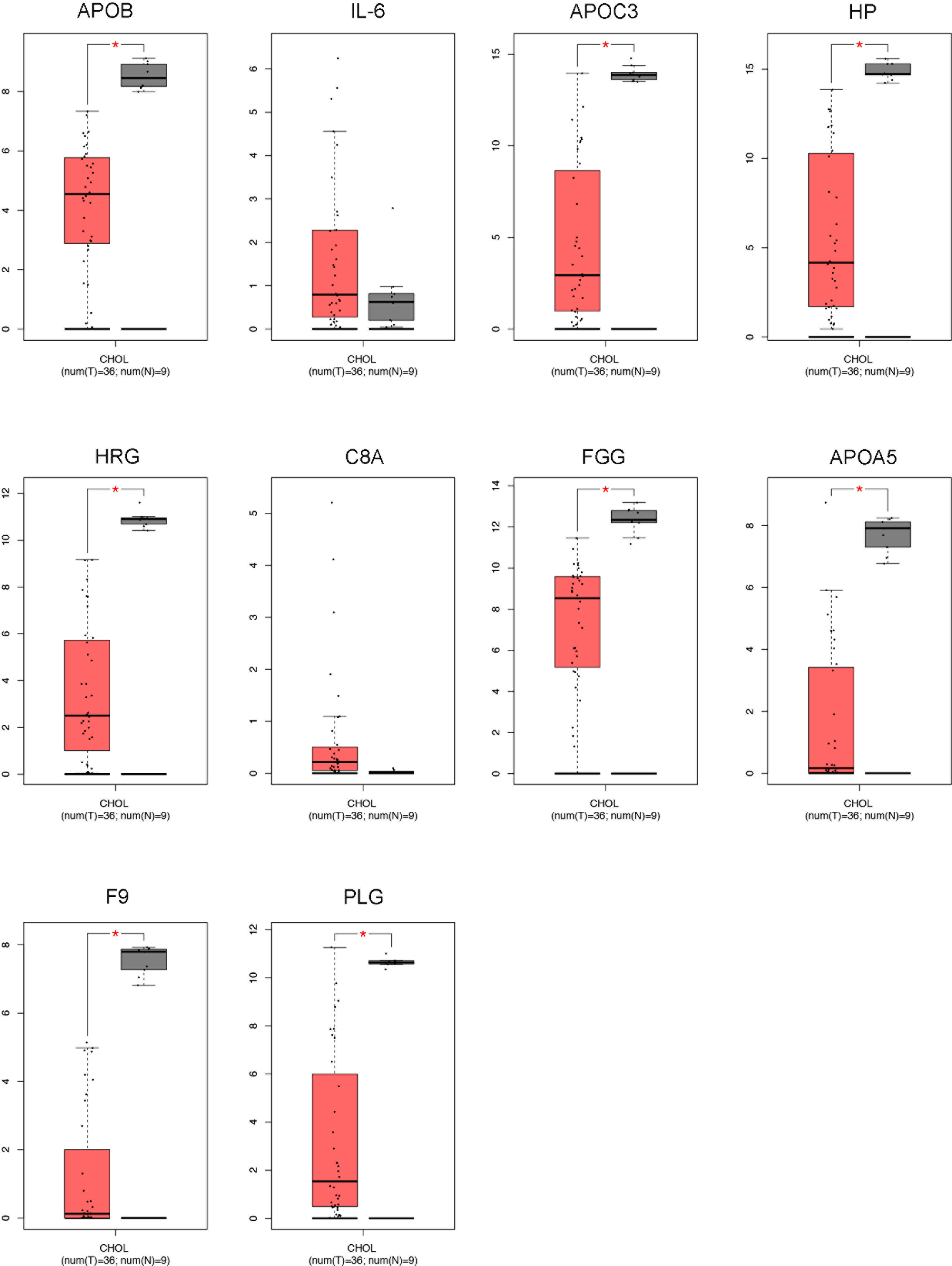

Supplement: Supplementary Figure 1 — Using GEPIA tools, these results indicated that APOB, APOC3, HP, HRG, FGG, APOA5, F9, and PLG were significantly down-regulated in CCA tissues compared to normal liver tissues. [file Image_1.tif]

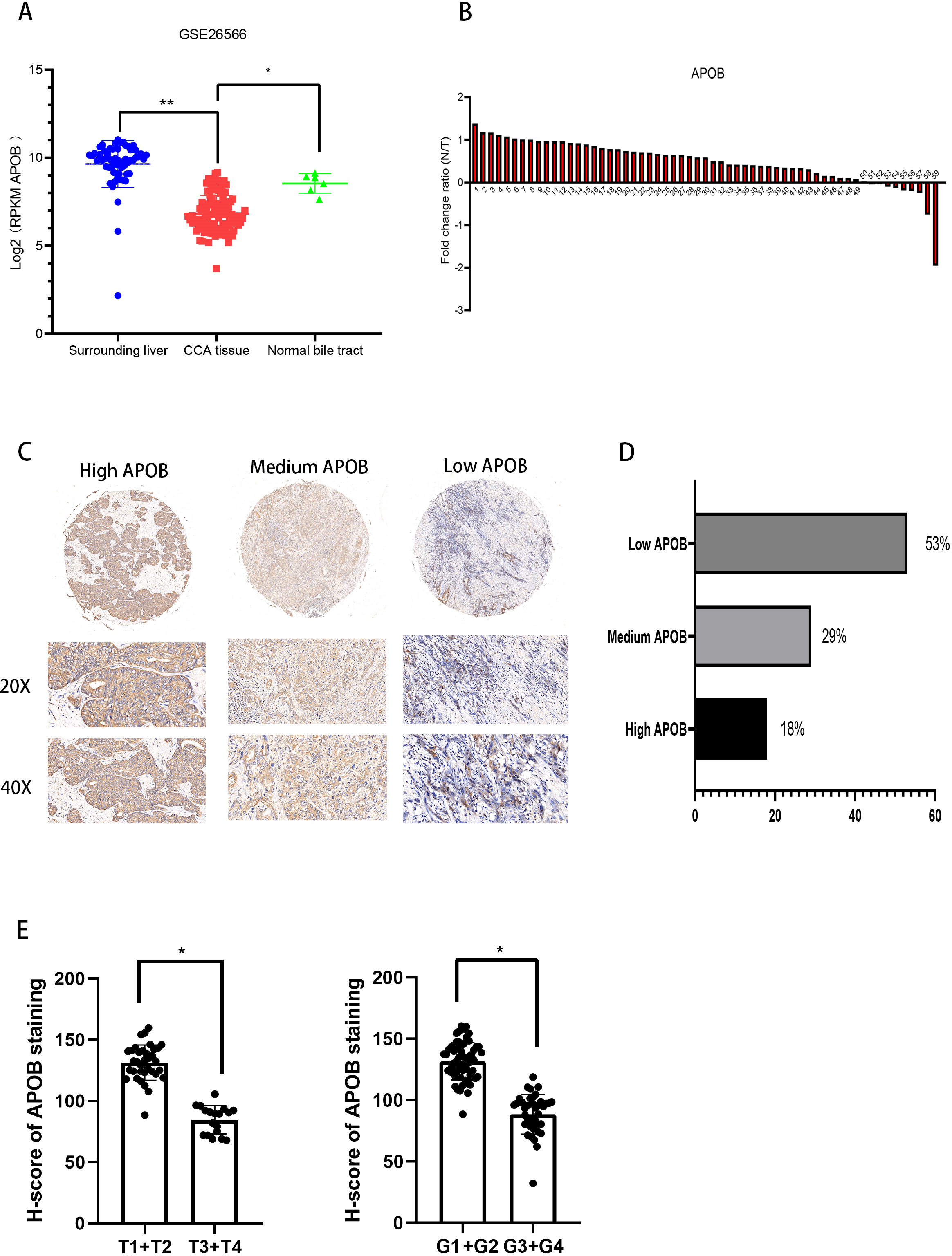

Supplement: Supplementary Figure 2 — (A, B) According to the GSE26566, we identified APOB was down-regulated in CCA tissues compared to the normal liver tissues counterparts. (C, D) The immunohistochemistry staining of APOB was performed in the CCA tumor microarray, about 53% of the cases presented the low expression of APOB, and 29% and 18% of the CCA tissues showed the medium and high expression of APOB respectively. (E) based on the TNM stage level, our results revealed that the APOB expression level of the CCA tissues in T3+T4 group was lower than that in T1+T2 group. Besides, we also identified that, followed by the increase of pathological grade, the expression of APOB reduced. [file Image_2.tif]

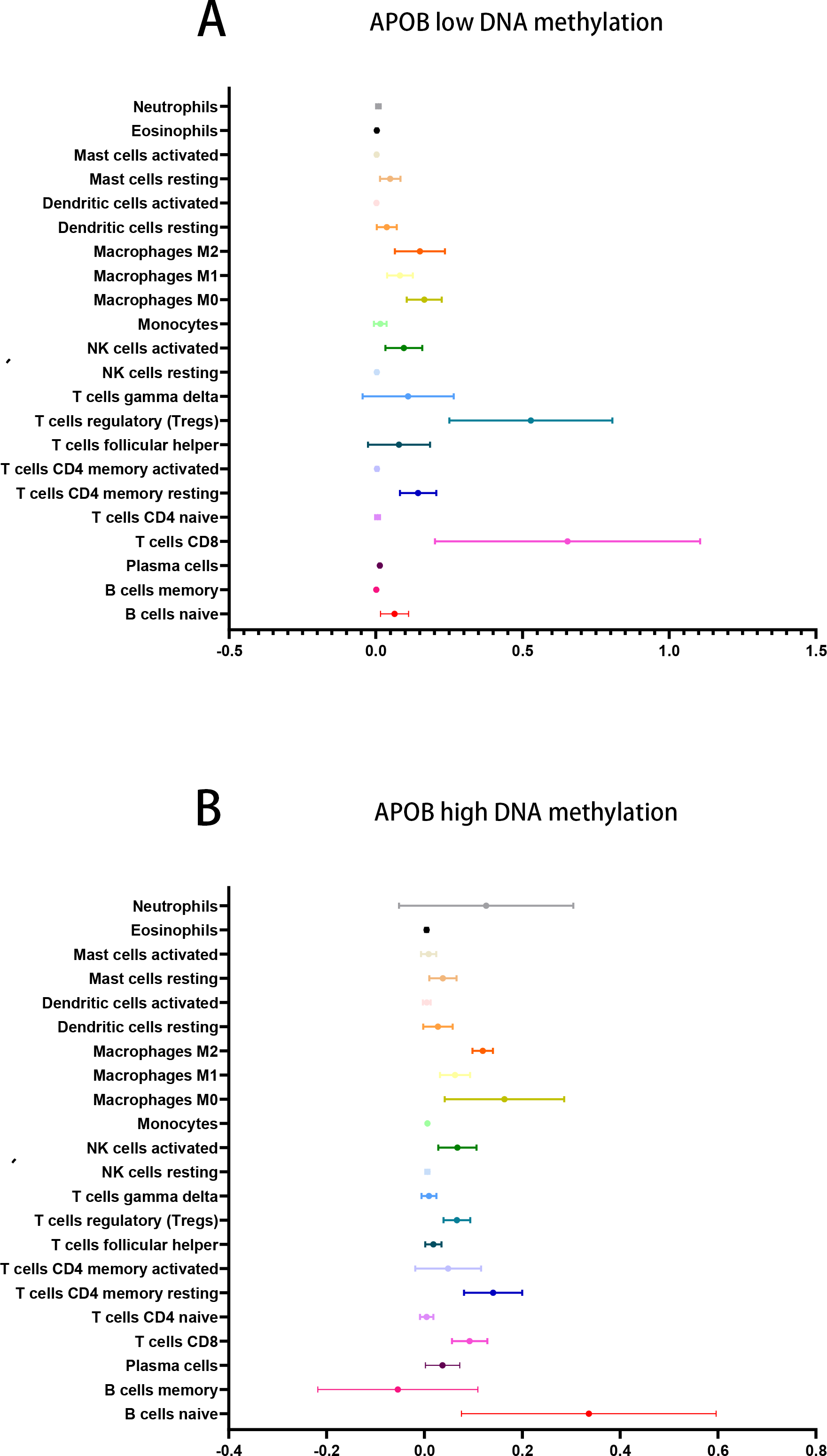

Supplement: Supplementary Figure 3 — Based on the MEDIP sequence data, the results showed that followed by the increase of APOB DNA methylation level, the infiltration of neutrophils and B cell naive were accelerated, but the infiltration of regulatory T cells and CD8+ T cells were inhibited in CCA. [file Image_3.tif]
